# Locus Coeruleus Norepinephrine in Learned Behavior: Anatomical Modularity and Spatiotemporal Integration in Targets

**DOI:** 10.3389/fncir.2021.638007

**Published:** 2021-06-07

**Authors:** Vincent Breton-Provencher, Gabrielle T. Drummond, Mriganka Sur

**Affiliations:** Picower Institute for Learning and Memory, Department of Brain and Cognitive Sciences, Massachusetts Institute of Technology, Cambridge, MA, United States

**Keywords:** locus coeruleus, noradrenaline (norepinephrine), neuromodulation, learning, inhibition, arousal, learned behavior

## Abstract

The locus coeruleus (LC), a small brainstem nucleus, is the primary source of the neuromodulator norepinephrine (NE) in the brain. The LC receives input from widespread brain regions, and projects throughout the forebrain, brainstem, cerebellum, and spinal cord. LC neurons release NE to control arousal, but also in the context of a variety of sensory-motor and behavioral functions. Despite its brain-wide effects, much about the role of LC-NE in behavior and the circuits controlling LC activity is unknown. New evidence suggests that the modular input-output organization of the LC could enable transient, task-specific modulation of distinct brain regions. Future work must further assess whether this spatial modularity coincides with functional differences in LC-NE subpopulations acting at specific times, and how such spatiotemporal specificity might influence learned behaviors. Here, we summarize the state of the field and present new ideas on the role of LC-NE in learned behaviors.

## Introduction

Norepinephrine (NE) is one of the four main neuromodulators in the brain, exerting widespread influence over almost all cortical and subcortical brain regions. Neurons in the locus coeruleus (LC) release NE to regulate baseline arousal and to facilitate a variety of sensory-motor and behavioral functions (Aston-Jones and Cohen, 2005; Sara, 2009; Sara and Bouret, 2012; Poe et al., 2020). Dysfunction in the LC-NE system has been implicated in the etiology of ADHD (Arnsten, 2011), schizophrenia (Friedman et al., 1999), anxiety or stress (Valentino and Van Bockstaele, 2008; Arnsten et al., 2015; Reyes et al., 2015), and depression (Delgado and Moreno, 2000), as well as in the cognitive decline observed in aging and Alzheimer’s disease (Weinshenker, 2008).

Despite its brain-wide effects and established involvement in CNS disorders, much about even the normal function of the LC-NE system in the brain remains unknown. For example, the conditions under which LC-NE neurons are transiently activated and the modes of NE action during learned behaviors are poorly understood, especially in comparison to other neuromodulatory systems, such as dopamine (Schultz et al., 1997; Schultz, 1998, 2016; Engelhard et al., 2019). There are at least three reasons for this gap. First, the activation of phasic NE has been traditionally examined in the context of sensory, motor, or goal-oriented events with little consideration of its role in signaling temporal stimulus-action-reward sequences or task parameters such as uncertainty. Second, the functions of phasic NE have been interpreted in the context of global or brain-wide influences, without considering selective spatial targeting. Third, the LC is a small nucleus, with a small number of neurons projecting throughout the brain, thus exhibiting relatively low innervation density as compared to other neuromodulatory systems, which typically have a larger number of neurons with a distinct projection network (e.g., substantia nigra and ventral tegmental area for dopamine). For this reason, methods for recording and manipulating LC-NE activity during behavior have been technically challenging. Recent studies have begun to overcome many of these challenges, enabling a more thorough understanding of the role of LC-NE in different aspects of cognition and behavior. Here, we review the current state of the field and present new ideas on the organization, activation, and function of LC-NE neurons.

## Anatomy of the Ne System

**FIGURE 1:**
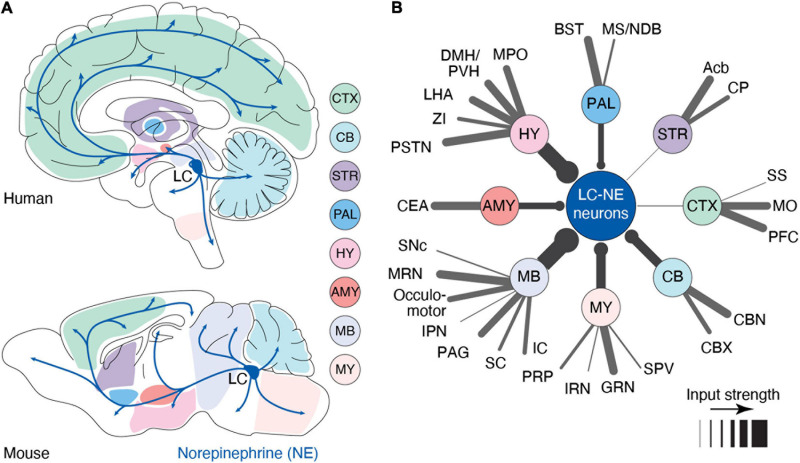
Anatomy of the LC-NE system. **(A)** Anatomy of the outputs originating from the LC nucleus in human and mouse. Shaded areas indicate major sub-regions that potentially send input to LC. In this illustration, we have assumed that input regions identified in mouse are similar in humans **(B)** Distal inputs to LC-NE neurons obtained by retrograde tracing using rabies virus targeted at LC-NE neurons in mice. Input regions are grouped by: cortex (CTX), striatum (STR), pallidum (PAL), hypothalamus (HY), amygdala (AMY), midbrain (MB), medulla (MY), and cerebellum (CB). The thickness of each line represents the strength of the input from each region. Input strength was calculated by counting the number of cells retrogradely labeled in a specific area and dividing it by the total number of retrogradely labeled neurons. Regions providing less than 0.5% of inputs were left out of this diagram. Local inputs from the pons were also excluded. PFC, prefrontal cortex; MO, motor area; SS, somatosensory area; Acb, nucleus accumbens; CP, caudoputamen; BST, bed nucleus of stria terminalis; MS/NDB, medial septal/diagonal band nucleus; MPO, medial preoptic area; DMH/PVH, dorsomedial/paraventricular nucleus; LHA, lateral hypothalamic area; ZI, zona incerta; PSTN, parasubthalamic nucleus; CEA, central amygdala; SNc, substantia nigra; MRN, midbrain reticular nucleus; IPN, interpeduncular nucleus; PAG, periaqueductal gray; SC, superior colliculus; IC, inferior colliculus; PRP, nucleus prepositus; IRN, intermediate reticular nucleus; GRN, gigantocellular reticular nucleus; SPV, spinal nucleus of the trigeminal; CBX, cerebellar cortex; and CBN, cerebellar nuclei. Data in **(B)** from Breton-Provencher and Sur (2019).

Despite its small number of neurons [∼3,000 neurons in the rodent brain (Sara, 2009) and ∼50,000 in the human brain (Sharma et al., 2010)], the LC serves as the primary source of NE in the forebrain with a highly divergent set of projections. Indeed, most cortical and subcortical areas receive dense LC-NE axonal innervation with the marked exception of the striatum (Pickel et al., 1974; Jones and Moore, 1977; Nomura et al., 2014), which receives limited innervations from the LC (Zerbi et al., 2019; Figure 1A). LC is reported to receive inputs from up to 111 distinct brain regions, including most brain-stem and forebrain regions (Schwarz et al., 2015; Breton-Provencher and Sur, 2019; Figure 1B). Brainstem inputs, most notably from the gigantocellular reticular nucleus (Jones and Yang, 1985) – which responds to tactile, visual, vestibular, and olfactory stimuli (Tabansky et al., 2018) – result in LC activation following salient sensory stimuli. Meanwhile, top-down inputs from the prefrontal cortex (PFC) and central amygdala can modulate the intensity of LC activation (Sara and Hervé-Minvielle, 1995; Jodo et al., 1998; McCall et al., 2015). These convergent inputs suggest its recruitment in both bottom-up, sensory induced, as well as top-down, goal-directed, regulation of behavior. This input-output organization leaves the LC poised to modulate brain function in response to external stimuli and internal states, and modify it through learning. Determining the contexts that trigger LC activity and how, in turn, this activity affects brain circuits is critical to understanding the role of LC-NE in cognition.

## Local LC Circuits

**FIGURE 2:**
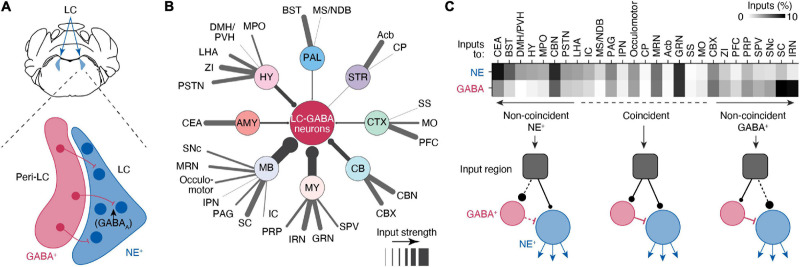
Circuits controlling local inhibition of LC-NE+ neurons. **(A)** Illustration of a coronal view of the LC and medial pericoeruleus area showing the location of LC-GABA and -NE neurons. **(B)** Distal inputs to LC-GABA neurons obtained by retrograde tracing using rabies virus targeted at LC-NE neurons. Input regions are grouped by: cortex (CTX), striatum (STR), pallidum (PAL), hypothalamus (HY), amygdala (AMY), midbrain (MB), medulla (MY), and cerebellum (CV). The thickness of each line represents the strength of the input from each region. Input strength was calculated by counting the number of cells retrogradely labeled in a specific area and dividing it by the total number of retrogradely labeled neurons. Regions providing less than 0.5% of inputs were left out of this diagram. Local inputs from the pons were also excluded. **(C)** Comparison between the input strength to LC-NE versus LC-GABA for all distal brain regions targeting the LC area. The darkness of each square in the top graph represents the fraction of input each region contributes to total input. Regions are divided between three types depending on whether they send coincident or non-coincident inputs to LC-NE or LC-GABA neurons. See Figure 1 for a list of abbreviations. Data in **(B,C)** from Breton-Provencher and Sur (2019).

Norepinephrine neuronal bodies make up a dense LC “core,” with LC-NE dendrites extending into a pericoeruleus “shell” region. The pericoerulear region also contains GABAergic (LC-GABA), glutamatergic, neuropeptide-S-expressing, and cholinergic neurons (Xu et al., 2004; Boucetta et al., 2014; Cox et al., 2016). Whether all these neuronal subtypes make direct connections with LC-NE neurons, as well as their function in LC modulated behaviors, remains unresolved. Nonetheless, the neuronal subtype that has received the most attention is the LC-GABA population, which forms inhibitory synapses on LC-NE neurons (Aston-Jones et al., 2004; Jin et al., 2016; Breton-Provencher and Sur, 2019; Kuo et al., 2020; Figure 2A). Tracing experiments indicate that these LC-GABA neurons receive inputs from largely the same brain regions as LC-NE neurons, with only several regions exhibiting preferential projections to either LC-GABA or LC-NE neurons (Breton-Provencher and Sur, 2019; Figures 2B,C). Direct recordings from LC-GABA neurons show that they are highly active during wakefulness (Cox et al., 2016; Breton-Provencher and Sur, 2019). A subpopulation of LC-GABA neurons are active simultaneously with LC-NE neurons, reflecting coincident inputs to both LC-NE and LC-GABA populations (Breton-Provencher and Sur, 2019). Coincident inputs to LC-NE and LC-GABA neurons play a critical role in controlling the phasic activity of LC-NE neurons (Kuo et al., 2020) and resultant transient increases in arousal in response to salient sensory stimuli (Breton-Provencher and Sur, 2019). Based on retrograde labeling, LC-GABA neurons would receive preferential, or non-coincident, inputs from several brain regions, suggesting that these regions can exert inhibitory control of LC-NE activity (Breton-Provencher and Sur, 2019; Figure 2C). Further studies using input mapping with electrophysiology are required to confirm the existence of preferential inputs to LC-GABA neurons. Yet, preferential inputs to LC-GABA neurons have been reported in previous experiments studying PFC inputs. Inactivation of the PFC in rats shows an increase in firing rate in the LC (Sara and Hervé-Minvielle, 1995), and axonal photo-activation of PFC inputs in the LC decreases baseline LC-mediated arousal levels (Breton-Provencher and Sur, 2019). Based on tracing experiments (Figure 2C), there are several other regions that likely exert preferential inputs to LC-GABA neurons, such as the superior colliculus or the intermediate reticular nucleus, but the function and nature of these input regions on the LC have been largely unexplored.

Whether the other neuronal subtypes in the LC shell – e.g., glutamatergic, neuropeptide-S-expressing, or cholinergic – form direct connections with LC-NE neurons, or play a role in shaping LC-NE activity, remains unknown. However, these neuronal subtypes are predominantly active during wakefulness (Boucetta et al., 2014; Cox et al., 2016), indicating that they could be involved in spontaneous or learned behaviors. Future research using physiological recordings and manipulations targeting these different neuronal cell-types will enable a more comprehensive understanding of LC local circuits.

## LC-NE Regulation of Arousal and Attention

One of the strongest behavioral correlates of LC-NE activity is its link with vigilance states. LC-NE neurons are silent during REM sleep, display low levels of activity during non-REM sleep, and are most active during wakefulness (Aston-Jones and Bloom, 1981a; Swift et al., 2018; Hayat et al., 2020). Manipulating LC activity modulates vigilance state. High levels of photo-activation of LC-NE neurons, mimicking LC phasic activity, promotes sleep-to-wake transitions (Carter et al., 2010), while lower photo-activation levels, resembling increased tonic activity, impair the power of theta and delta frequencies measured with EEG during sleep (Swift et al., 2018). Similarly, minimal photo-activation of tonic LC-NE increases the probability of sensory-evoked awakenings (Hayat et al., 2020). On the other hand, reducing LC-NE activity with photo-inactivation decreases wakefulness durations (Carter et al., 2010). Together, these results suggest that LC-NE plays an important role in controlling vigilance states, likely as part of a complex network involving neurons from the brainstem, mid-brain, thalamus, and hypothalamus (Berridge, 2008; Lee and Dan, 2012; Gent et al., 2018).

The link between LC-NE activity and arousal is likely relevant for explaining the role of LC-NE in learned behaviors during wakefulness. LC activation tightly correlates with pupil size and arousal level in mice (Reimer et al., 2016; Breton-Provencher and Sur, 2019), monkeys (Joshi et al., 2016), and humans (Murphy et al., 2014; Elman et al., 2017; DiNuzzo et al., 2019). This activity of LC-NE neurons alone is sufficient to promote global arousal in mice, as photo-activation or -inhibition of LC-NE neurons during wakefulness increases or reduces arousal levels, respectively (Carter et al., 2010; Lovett-Barron et al., 2017; Breton-Provencher and Sur, 2019; Hayat et al., 2020). The mode of activation of LC-NE neurons seems to play a key role in arousal. Indeed, phasic activation over long periods (>1 h), but not tonic activation, of LC-NE activity increases arousal (Carter et al., 2010). Change in arousal can occur following phasic LC-NE activation by salient or novel sensory stimuli (Aston-Jones and Bloom, 1981b; Grant et al., 1988; Vankov et al., 1995; Takeuchi et al., 2016). Often, this phasic activation scales with the strength of behavioral action associated with the stimulus (Grant et al., 1988; Breton-Provencher and Sur, 2019). Optimal cognitive performance correlates with moderate levels of baseline LC-NE activity, while uncontrolled LC-NE activity can lead to hyper-arousal and anxiety behaviors (Valentino and Van Bockstaele, 2008; McCall et al., 2015, 2017; Li et al., 2018), exemplifying the inverted-U relationship between brain functions and NE activation (Yerkes and Dodson, 1908; Usher et al., 1999; Aston-Jones and Cohen, 2005; McGinley et al., 2015).

Arousal alters attention by suppressing low-salience stimuli while enhancing responses to highly salient or goal-directed information; accordingly, LC-NE is also thought to be critically involved in regulating attention through its brain-wide release (Lee et al., 2018). Older adults typically exhibit a decline in LC functional connectivity that correlates with an inability to suppress non-salient information during arousal (Lee et al., 2018). Global LC-mediated changes in arousal can alter attention via gain control, though the mechanism by which this gain control occurs remains unresolved. It has been shown that pharmacological blockade of NE activity impairs the membrane depolarization of cortical neurons that would naturally occur during high levels of arousal (Constantinople and Bruno, 2011; Polack et al., 2013; Schiemann et al., 2015). Arousal levels have also been shown to modulate microglial response to brain injury via β2 adrenergic receptors (Stowell et al., 2019). However, a detailed analysis of the cell types and neuronal subtypes being affected by NE during brain state changes is lacking. Moreover, we have a poor understanding of the degree of brain-wide modulation by LC-NE activity *in vivo* in awake behaving animals. Finally, defining the function of LC-NE activity as a global broadcast system of arousal might be too simplistic for understanding its role in learned behavior, as we discuss later with the emergence of modular functional data in the LC-NE system.

## Activation of LC-NE Neurons During Learned Behavior

The role of LC-NE neurons extends beyond arousal, attention, and anxiety. Recordings or pharmacological manipulations of phasic NE activity suggest a dual involvement in behavior and cognition. First, LC activation is critical for sustained attention, as its phasic activity correlates with behavioral responses in learned behaviors (Bouret and Sara, 2004; Clayton et al., 2004; Rajkowski et al., 2004; Bouret and Richmond, 2009, 2015; Kalwani et al., 2014; Varazzani et al., 2015; Jahn et al., 2020). Non-targeted recordings in the LC show a transient increase in firing rate coinciding with behavioral response in tasks involving operant or instrumental conditioning. This increase prior to motor execution scales with the amount of effort or vigor exerted (Rajkowski et al., 2004; Varazzani et al., 2015), but also with expected reward size (Bouret and Richmond, 2015). During periods of poor behavioral performance, LC activity is decreased. Thus, it has been hypothesized that transient LC activity during a learned behavior is critical for maintaining focused attention on a task. Supporting this hypothesis, long-lasting blockade of LC activity slows reward seeking behaviors (Jahn et al., 2018), and pairing LC activity with a sensory stimulus improves this response (Martins and Froemke, 2015). Though there is abundant evidence for LC-NE activity facilitating behavioral execution, how this is achieved, especially in the context of other LC-NE functions, is an outstanding question in the field.

In addition to its role in facilitating behavioral execution, LC activation is critically involved in learning and memory. LC projections to the hippocampus, amygdala, and PFC influence spatial learning and memory formation (Kempadoo et al., 2016; Takeuchi et al., 2016; Uematsu et al., 2017; Wagatsuma et al., 2017; Kaufman et al., 2020). For example, during passive reinforcement learning, LC axonal activity in the hippocampus is associated with the timing of reward in spatial memory encoding (Kaufman et al., 2020). In tasks requiring active learning, LC activity correlates with an animal’s ability to form new stimulus/response associations: attentional set-shifting paradigms show that LC activity is involved in the learning of a new discrimination and in making extra-dimensional shifts (Devauges and Sara, 1990; Tait et al., 2007; Janitzky et al., 2015; Cope et al., 2019; Glennon et al., 2019). In rats performing a linear maze navigation task, when animals undergo an extra-dimensional shift requiring them to use visual cues instead of the previously indicative spatial cues, boosting NE pharmacologically accelerates the detection of the cue-shift and learning of a new cue (Devauges and Sara, 1990). It has also been shown that in rats performing a task requiring a shift in association to a new sensory cue within the same modality (intra-dimensional shift), LC spiking activity precedes prelimbic cortex spiking activity, suggesting that NE plays a critical role in the updating of task rules (Bouret and Sara, 2004). Further, decreasing LC-NE activity in the PFC over long durations with chemogenetics impairs a rat’s ability to disengage from a cognitive task (Tervo et al., 2014; Kane et al., 2017).

One mechanism by which LC-NE activity could enable learning is through encoding of environmental novelty. In naïve animals, electrophysiological measurements show that LC activity increases in response to novel or salient stimuli (Hervé-Minvielle and Sara, 1995; Vankov et al., 1995; Takeuchi et al., 2016; Breton-Provencher and Sur, 2019), and this activity quickly habituates (Hervé-Minvielle and Sara, 1995; Vankov et al., 1995). In trained animals, non-targeted measurements of LC spiking activity during learning of an operant conditioning task shows transient LC activity linked with unexpected reward (Bouret and Sara, 2004). Indeed, transient increase in LC activity may represent “unexpected uncertainty” and serve as a neural interrupt signal for unexpected events (Yu and Dayan, 2005; Dayan and Yu, 2006). However, it remains unclear whether increased LC activity in response to unexpected stimuli is causal for learning mechanisms, and how this might co-exist with its role in behavioral execution.

## LC-NE Activity in Human Cognition

It has been widely observed that LC dynamics correlate with fluctuations in pupil diameter, both in human and animal studies (Murphy et al., 2014; Joshi et al., 2016; Reimer et al., 2016; Breton-Provencher and Sur, 2019). Many studies have taken advantage of this correlation to study the role of the LC in arousal (Krishnamurthy et al., 2017), learning (Nassar et al., 2012), and uncertainty (Urai et al., 2017) in human subjects. One such study suggests that LC-NE supports learning through global modulations of neural gain (Lee et al., 2018). High neural gain would enhance attention for selective stimulus features, whereas low gain would broaden attention, promoting processing of multiple stimulus features (Eldar et al., 2013; Lee et al., 2018). fMRI data show that periods of high gain and focused learning are associated with tightly clustered functional connectivity, suggesting that increased gain enhances selective interactions in brain networks (Lee et al., 2018). In addition to the evidence for LC-NE activity modulating feature processing through changes in the gain of global networks, other experiments have implicated LC-NE in learning through driving behavioral responses to uncertainty and volatility (Krishnamurthy et al., 2017; Urai et al., 2017; Lawson et al., 2021). Indirect measurements of NE activity in humans using pupillometry report a correlation between unexpected outcomes and increases in transient LC activity (Browning et al., 2015). Experiments using human subjects performing a probabilistic learning task show that blocking NE binding to β-adrenergic receptors globally causes subjects to rely more on their prior expectations when uncertainty is high (Lawson et al., 2021). Similarly, in a study using an auditory localization task where the predictability of the location varied over time, arousal was found to adjust the strength of perceptual biases in a changing environment (Krishnamurthy et al., 2017). These studies again highlight the potential roles of LC-NE in human cognition: driving attention and behavioral execution, and facilitating learning through updating of priors. Although pupil dilations strongly correlate with LC-NE activity, other neuromodulators such as acetylcholine and serotonin also show some degree of correlation with pupil size (Reimer et al., 2016; Larsen and Waters, 2018; Cazettes et al., 2021). Further tools to investigate neuromodulator dynamics will be required to disentangle the role of LC-NE from that of other neuromodulators in human behaviors.

## Theories of LC Function

Two predominating theories attempt to explain the role of the LC in sensory-motor behavior: the adaptive gain theory (Aston-Jones and Cohen, 2005) and the network reset theory (Bouret and Sara, 2005; Yu and Dayan, 2005; Dayan and Yu, 2006). The adaptive gain theory seeks to explain the phasic and tonic modes of LC-NE activity. Phasic activity prevails during optimal behavioral performance, where transient increases in LC-NE activity facilitate task-specific decision processes (Usher et al., 1999; Aston-Jones and Cohen, 2005). In contrast, tonic activity prevails during periods of poor performance, where a general increase in LC-NE activity increases the gain of a network indiscriminately, making targeted circuits more responsive to any stimulus (Usher et al., 1999; Aston-Jones and Cohen, 2005). Thus, through adaptive gain, LC-NE activity optimizes the tradeoff between exploitation and exploration behaviors by switching between phasic and tonic activity, respectively. However, this theory does not explain which environmental stimuli would cue the LC to switch between these two modes. Further, this theory does not offer an explanation for whether different phasic activities exist temporally within tasks, and what the roles of these temporally distinct phasic activations might be. The network reset theory, on the other hand, suggests that contexts requiring a change in behavior transiently activate LC-NE neurons (Bouret and Sara, 2005; Yu and Dayan, 2005; Dayan and Yu, 2006). These activating contexts lead LC-NE neurons to induce widespread cortical arousal and reset network activity in the brain. Similarly, it has been suggested that LC-NE activity signals “unexpected uncertainty,” causing a reset in network activity to enable an updating of priors (Yu and Dayan, 2005; Dayan and Yu, 2006). By signaling the need to update priors, LC-NE would suppress top-down, expectation driven information in favor of bottom-up sensory-induced signals to enable learning and behavioral optimization. However, it is not clear how LC responses during execution would not lead to a network reset, and how phasically induced arousal relates to the commonly described tonic regulation by NE of arousal and internal state.

Though these theories of gain-modulation and network reset are not mutually exclusive, neither alone can fully explain the role of LC-NE in the brain, and little progress has been made in either refining or unifying them in the past 15 years. Recent technological advances and increased tool availability enabling precise measurements and manipulations of LC spiking activity in awake behaving mice present a means of evaluating these theories or advancing alternative proposals. Future studies on the role of LC-NE in learned behavior will require well-designed behavioral experiments to address these dual functions, while also considering the heterogeneous nature of LC activity and potential for spatial modularity of outputs.

## Modular Outputs of the LC

One way to reconcile the divergent roles for LC-NE activity is suggested by recent evidence of spatial modularity within the LC-NE neuronal population (Chandler et al., 2019; Foote and Berridge, 2019; Poe et al., 2020). Historically, the LC has been considered a homogenous nucleus, such that NE is uniformly released to forebrain, brainstem, cerebellum, and spinal cord (Aston-Jones and Waterhouse, 2016). Yet, retrograde labeling shows that morphological characteristics and location within the LC nucleus is predictive of the particular region a LC neuron innervates (Loughlin et al., 1986a,b). Recent findings using projection-based viral genetic labeling has confirmed this view (Schwarz et al., 2015; Hirschberg et al., 2017; Uematsu et al., 2017; Plummer et al., 2020; Poe et al., 2020). Retrograde labeling of LC-NE neurons from the infralimbic, prelimbic, motor cortices or the amygdala, olfactory bulb, and medulla yields very few axons in other regions; these data suggest modularity in NE neuron outputs (Hirschberg et al., 2017; Uematsu et al., 2017; Plummer et al., 2020; Poe et al., 2020). However, other reports using the same approach demonstrate broad outputs from the LC, with broad collateralization of LC-NE axons in most brain regions excluding the olfactory bulb and medulla (Schwarz et al., 2015). Retrograde tracings also demonstrate limited modularity of LC-NE axons projecting to primary sensory cortices (Kim et al., 2016). Moreover, a high throughput method for mapping projections from the LC shows that LC neurons can preferentially target one cortical area, but also innervate large areas of cortex at reduced density (Kebschull et al., 2016). LC-NE neurons may also display functional modularity: simultaneous recording of LC neuron activity in anesthetized rats shows ensembles where the activity of subgroups of neurons correlates in time (Totah et al., 2018). So far, no data exist on whether functional modularity exists in awake behaving animals and whether this modularity originates from a combination of distant and local (e.g., LC-GABA) inputs.

**FIGURE 3:**
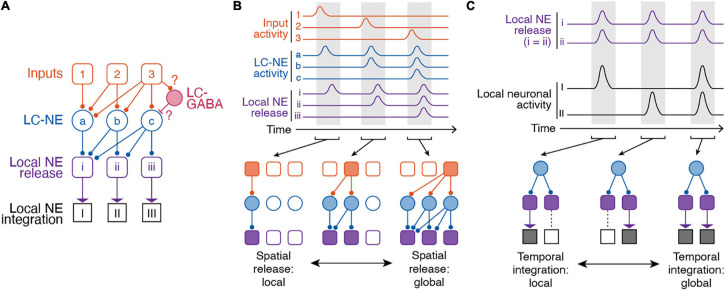
Spatiotemporal dynamics of the LC-NE system. **(A)** Anatomical organization of inputs to and outputs of LC. Note, LC-GABA neurons were included in this illustration as potential mechanism for nuancing local LC-NE activity, but, so far, no data exist on the relationship between local LC-GABA and specific LC-NE outputs. **(B)** Spatial modularity of LC-NE release. Top – Example activity of inputs to LC-NE neurons (orange), LC-NE neurons (blue), and local LC-NE release (purple). Bottom – Local versus global release of NE in output regions is dependent on which LC-NE neurons are activated by a given input. **(C)** Temporal modularity of LC-NE neuromodulation. Top – activity of local NE release in two given output regions and underlying neuronal activity in each region. Note, we assume that NE release is spatially global for simplicity. We also assume that NE neuromodulation increases neuronal activity in both target regions. Bottom – Due to differential NE receptor expression in brain regions, heterogenous expression of NE receptors on different types of brain cells, or the underlying function of a specific brain region, temporal integration can be local or global.

Our current understanding of the LC-NE circuitry suggests at least partially distinct populations of LC-NE neurons with respect to their projection targets and functional organization (Figure 3A). These distinct LC-NE neurons would be able to confer target specific NE release through spatial modularity. In this spatial model of modularity, subpopulations of LC-NE neurons could receive different types of inputs. Distinct inputs to LC-NE neuron with highly convergent axons would enable local NE release (Figure 3B). In other behavioral contexts, inputs to LC-NE neurons with highly divergent axons, or global inputs to most LC-NE neurons, would enable global NE release (Figure 3B). Distinct outputs from LC neurons to cortical or subcortical targets would then encode distinct behavioral correlates. These proposals thus represent testable hypotheses of LC function in learned behaviors derived from spatial modularity of its inputs and outputs.

## Molecular Heterogeneity in the LC

The suggestion of a modular input-output organization in the LC raises the question of whether LC-NE subpopulations exhibit different molecular signatures that could confer different functional properties. Again, though the LC has often been considered a homogenous nucleus, there is evidence to suggest that LC-NE neurons are a molecularly heterogeneous population. For example, though all LC neurons contain NE, separable subpopulations also contain other neuropeptides, such as Neuropeptide Y, galanin, and cocaine-and-amphetamine-regulated transcripts (Holets et al., 1988; Koylu et al., 1999; Simpson et al., 1999; Devoto et al., 2001, 2005). These subpopulations are found throughout the LC but have biased distributions; however, the anatomy of the projections from these neurons as well as the functional relevance of this co-release is uncharacterized (Holets et al., 1988). Galanin release from LC has been suggested to mediate active-coping behaviors, but whether this release occurs in projection-specific LC neurons is not known (Tillage et al., 2020). It has also been shown that LC projections release the neuromodulator dopamine in the hippocampus (Kempadoo et al., 2016; Takeuchi et al., 2016; Wagatsuma et al., 2017) and midline thalamus (Beas et al., 2018), where it plays a significant role in memory consolidation and stress responses, respectively. The extent to which dopamine is co-released from LC terminals in other areas of the brain, and how this co-release might affect other behaviors, remains unknown. In addition to having subsets of neurons capable of co-release of neuropeptides and neurotransmitters, LC neurons themselves appear to exhibit variability in neurotransmitter receptor expression, potentially enabling subpopulations to be activated by different inputs. LC neurons express receptors for many different neurotransmitters, such as GABA, orexin/hypocretin, and acetylcholine (Egan and North, 1986; Luque et al., 1994; Mansour et al., 1994; Marcus et al., 2001). In addition to these receptors, LC-NE neurons express several adrenoceptor subtypes, making LC-NE neurons themselves responsive to NE release. Indeed, it has been suggested that these adrenoceptor subtypes are differentially expressed throughout the LC, making subpopulations of LC-NE neurons differentially responsive to LC-NE activation (Young and Kuhar, 1980; Chamba et al., 1991). Like many other aspects of LC organization and function, the role of these different molecularly defined subpopulations remains unexplored, but provides a means by which LC-NE subpopulations could be differentially activated to facilitate distinct aspects of learned behavior.

## Target-Specific Spatial and Temporal Effects of LC-NE Activity

Local integration of NE release by targets is an important way by which the activity of single LC neurons exhibiting multiple task components, with projections to multiple areas, can nonetheless be utilized for separate functions. Due to different NE receptor expression throughout the cortex (Goldman-Rakic et al., 1990; Schiemann et al., 2015), heterogenous expression of NE receptors on different types of brain cells (Hertz et al., 2010), or the underlying activity of a specific brain region, it is possible that the same NE signal can have different effects in output regions. Indeed, specific functions have been attributed to NE from region-specific pharmacological or optogenetic manipulations of LC activity. In subcortical regions, LC-NE activity in the hippocampus is critical for contextual memory formation (Wagatsuma et al., 2017) and place map plasticity (Kaufman et al., 2020). In the basolateral amygdala, photostimulating LC axons increases anxiety-like behavior (McCall et al., 2017). In the context of memory formation, inactivating LC projections to the basolateral amygdala impairs memory acquisition, while inactivating LC-NE projections in the infralimbic cortex impairs memory extinction (Uematsu et al., 2017). In cortical regions, pharmacological blockade of local NE receptors in visual, somatosensory, or motor cortex reduces membrane depolarization of neurons that is typically associated with state changes (Constantinople and Bruno, 2011; Polack et al., 2013; Schiemann et al., 2015). This depolarization in the presence of NE normally leads to an increase in firing rate of neurons and ultimately, increased sensory sensitivity (Polack et al., 2013). In prefrontal cortex regions such as the anterior cingulate cortex and limbic cortex, local manipulation of NE activity influences sustained attention (Berridge et al., 2012; Spencer and Berridge, 2019), memory (Berridge et al., 2012; Spencer and Berridge, 2019), decision making, and task acquisition (Tait et al., 2007; Tervo et al., 2014; Cope et al., 2019; Joshi and Gold, 2019). NE release in the anterior cingulate cortex promotes exploration and behavioral variation (Tervo et al., 2014). In a set-shifting task, manipulating NE activity in the prelimbic or infralimbic cortex improves the ability of animals to learn new associations (Tait et al., 2007; Cope et al., 2019). Importantly, most of these functions related to LC-NE activation are linked in time to specific components of internal state or behavior, so that NE release within a target enables the subsequent contribution of that target to brain state or function. Thus the different effects of LC-NE signals on processing and behavior not only reflect differential targeting of LC-NE outputs (spatial modularity) but also the differential processing of NE signals in time based on the functional role of the target as well as its regional and cellular NE receptor expression and availability (temporal modularity). In combination with spatial modularity, local and global temporal integration of NE signals in diverse targets is therefore an important mechanism of LC-NE function (Figure 3C).

## Discussion – Future Prospects

Understanding the complex role of LC-NE signals in facilitating distinct aspects of behavior will require understanding the timescales on which LC-NE neurons are activated, how this activity affects performance in a learned behavior, and how these LC-NE signals are used by target regions to facilitate behavioral responses and learning. As in human studies manipulating the discriminability of a target, a learned behavior for studying LC-NE in model systems should incorporate different levels of sensory uncertainty. Thus, a task dependent on trial-history that incorporates sensory uncertainty and necessitates a goal-directed behavioral choice would enable a more unified understanding of the role of LC-NE in learned behaviors. Another important goal for the field is developing an understanding of how NE interacts with other neuromodulators to modulate brain states and behaviors. An established task that can be used across neuromodulatory systems would be well-suited for this goal. For example, in the cholinergic system, an auditory go/no-go task was used to show that cholinergic neurons encode reinforcement surprise, and that these responses scale with uncertainty (Hangya et al., 2015). Using varying tone intensities, the difficulty was modulated on a trial-by-trial basis to reflect different degrees of uncertainty. A similar task might be used to study how LC-NE neurons encode task-relevant responses and uncertainty, all within the same framework. Additionally, a novel two-alternative forced choice task in head fixed mice has been used to assay perceptual and value-based decision making (Burgess et al., 2017; Steinmetz et al., 2019). This task, which has been standardized across laboratories, could be applied toward studying the role of LC in learned behaviors. Behaviors such as these, incorporating both behavioral execution and learning, combined with cutting-edge tools such as optogenetics, targeted labeling of neuronal populations, deep-brain imaging, and imaging of NE receptor dynamics, combined with specific hypotheses of LC-NE function as proposed here, will help elucidate the role of LC-NE in cognition.

## Conclusion

Despite the diverse roles of the LC in regulating arousal, attention, and facilitating more complex behaviors, our understanding of this nucleus is quite limited. Recent studies, however, are changing our understanding of the LC. What was formerly considered a homogenous nucleus exerting global, uniform influence over its many diverse target regions, is now suggested to be a heterogenous population of NE releasing cells, potentially exhibiting both spatial and temporal modularity that govern its function. These observations, combined with a rapidly expanding neuroscience toolkit, enable updating of existing theories, or potentially forming new ones, to explain the roles of LC-NE in learned behaviors.

## Author Contributions

VB-P and GD wrote the initial draft. VB-P made all figures. All authors discussed the content and commented on the text and figures.

## Conflict of Interest

The authors declare that the research was conducted in the absence of any commercial or financial relationships that could be construed as a potential conflict of interest.
